# Community Resilience Estimates and HIV Health Outcomes: Insights from the CNICS Cohort

**DOI:** 10.1007/s10461-025-04984-5

**Published:** 2025-12-16

**Authors:** L. Sarah Mixson, Stephanie A. Ruderman, Sonia Napravnik, Lindsay E. Browne, Deana M. Agil, Heather I. Henderson, Bridget M. Whitney, Mari M. Kitahata, Geetanjali Chander, Joseph A. C. Delaney, Heidi M. Crane, Carolyn A. Fahey

**Affiliations:** 1 Department of Medicine, University of Washington School of Medicine, 1959 NE Pacific Street, Seattle, WA 98195-6420 USA; 2 Division of Infectious Diseases, Department of Medicine, University of North Carolina School of Medicine, 130 Mason Farm Rd, 2101 Bioinformatics Building, Chapel Hill, NC 27599 USA

**Keywords:** Health disparities, Viral load, CD4 lymphocyte count, Community health

## Abstract

This study aims to better understand the association between community social vulnerabilities (e.g., income-to-poverty ratio, health insurance coverage) and the ability of people with HIV (PWH) to achieve viral suppression and optimal CD4 cell count levels. We conducted an observational study within the CFAR Network of Integrated Clinical Systems (CNICS) cohort of adult PWH in care at two CNICS sites between 1/2015 and 3/2023 with an identifiable residential address and HIV-related laboratory measures. Our exposure of interest, 2022 Community Resilience Estimates (CRE) from the US Census Bureau, is calculated at the census tract level and categorized as 0, 1–2, or 3–10 social vulnerabilities that impact community resilience. Outcomes included individual-level unsuppressed HIV viral load (≥ 50 copies/mL) and low CD4 count (< 350 cells/mm^3^). We estimated prevalence ratios and 95% confidence intervals (CI) using multivariable relative risk regression clustered by census tract, adjusting for individual-level demographic characteristics, site, and year of outcome measurement. Among 3,191 PWH, 342 (11%) were virally unsuppressed and 528 (17%) had low CD4 cell count. Compared to PWH in tracts with 0 vulnerabilities, those living in census tracts with 1–2 social vulnerabilities were 1.28 times more likely (95%CI: 1.00-1.63) to be virally unsuppressed. Those in census tracts with ≥ 3 social vulnerabilities were 1.95 times more likely (95%CI: 1.22–3.10) to be virally unsuppressed and were 1.51 times more likely (95%CI: 1.12–2.04) to have low CD4 cell counts. In the context of PWH, the CRE could be used to highlight areas for targeted interventions based on community-level influences to improve HIV-related outcomes.

**Supplementary Information:**

The online version contains supplementary material available at 10.1007/s10461-025-04984-5.

## Resumen

Este estudio tiene como objetivo comprender mejor la asociación entre las vulnerabilidades sociales de la comunidad (por ejemplo, la relación ingresos-pobreza, la cobertura del seguro médico) y la capacidad de las personas con VIH (people with HIV, PWH) para lograr la supresión vírica y unos niveles óptimos de recuento de linfocitos CD4. Realizamos un estudio observacional en la cohorte de la Red CFAR de Sistemas Clínicos Integrados (CNICS) de personas adultas con VIH que recibieron atención en dos centros de CNICS entre enero de 2015 y marzo de 2023, con un domicilio residencial identificable y medidas de laboratorio relacionadas con el VIH. Nuestra exposición de interés, las Estimaciones de Resiliencia Comunitaria (CRE) de 2022 de la Oficina del Censo de los EE. UU., se calcula a nivel de sector censal y se clasifica en 0, 1–2 o 3–10 vulnerabilidades sociales que afectan a la resiliencia comunitaria. Los resultados incluyeron la carga vírica del VIH no suprimida a nivel individual (≥ 50 copias/ml) y un recuento bajo de CD4 (< 350 células/mm^3^). Estimamos los cocientes de prevalencia y los intervalos de confianza (IC) del 95% utilizando una regresión de riesgo relativo multivariable agrupada por sector censal, ajustando las características demográficas a nivel individual, el centro y el año de medición de los resultados. Entre las 3191 PWH, 342 (11%) no tenían supresión vírica y 528 (17%) tenían un recuento bajo de linfocitos CD4. En comparación con las PWH en sectores con 0 vulnerabilidades, las que vivían en sectores censales con 1–2 vulnerabilidades sociales tenían 1,28 veces más probabilidades (IC del 95%: 1,00–1,63) de no tener supresión vírica. Las personas de sectores censales con ≥ 3 vulnerabilidades sociales tenían 1,95 veces más probabilidades (IC del 95%: 1,22 − 3,10) de no tener supresión vírica y 1,51 veces más probabilidades (IC del 95%: 1,12 − 2,04) de tener recuentos bajos de linfocitos CD4. En el contexto de las PWH, las CRE podrían utilizarse para destacar las áreas en las que se deben realizar intervenciones específicas basadas en las influencias a nivel comunitario para mejorar los resultados relacionados con el VIH.

## Background

Despite advances in HIV prevention and the availability of antiretroviral therapy (ART), communities characterized by high social vulnerabilities have disproportionately elevated rates of HIV diagnoses [1, 2]. Social vulnerability refers to the potential for adverse health effects within communities as a result of external stressors [3]. Community-level social vulnerabilities in the form of structural factors such as poverty, employment, and lack of broadband internet access may affect a community by reducing access to critical healthcare resources [4–8] and social support systems [9, 10]. These upstream determinants of health are unevenly distributed across communities due to systemic factors such as exclusionary housing practices [11, 12] that sort groups of people into different neighborhoods and perpetuate social and economic disadvantage [13], potentially shaping many individual-level health disparities for people with HIV (PWH) [14–16]. Socially vulnerable communities may also have less capacity to address common challenges faced by PWH such as stigma [17], discrimination [18], and mental health issues [19], further hindering individuals’ ability to adhere to HIV treatment [7, 20, 21] and achieve viral suppression [7, 22–24].

Community stakeholders and policymakers utilize various metrics to assess the potential vulnerability and resiliency of their communities [25]. Community resilience is the capacity of communities to withstand, adapt to, and recover from adversity [26]. This concept has gained attention as an emerging critical framework for understanding health disparities experienced by PWH [27, 28], especially in the context of upstream social vulnerabilities. Resilience is influenced by the social, economic, and environmental attributes of a community [29, 30]. Factors that impact a community’s vulnerability to disasters and public health emergencies, including income-to-poverty ratio, caregiver availability, household crowding, and communication barriers, highlight the intersection between resilience and social vulnerabilities.

While individual-level SES is well understood to impact HIV outcomes [5, 31], little is known about the broader community-level factors that influence health outcomes among PWH [27]. In this study, we used a community resilience index, which includes several social vulnerability indicators, and individual-level clinical data to examine the association between social vulnerability and unsuppressed HIV viral load (VL) and low CD4 cell count. We hypothesized that PWH living in census tracts with more social vulnerabilities would have worse HIV-related clinical outcomes.

## Methods

### Setting

The Centers for AIDS Research Network of Integrated Clinical Systems (CNICS) is a prospective, observational cohort study of PWH ≥ 18 years of age receiving routine clinical care at a network of medical centers across the US [32]. Two CNICS sites that had available geocoding data for their participants at the time of this study were included: University of North Carolina at Chapel Hill (UNC), which is located in a suburban area, and University of Washington (UW) in Seattle, a densely populated urban area. These two sites provide opportunities to examine how community-level social vulnerabilities may impact HIV outcomes in different geographic contexts.

The CNICS data repository integrates comprehensive data from both outpatient and inpatient encounters. Demographic, clinical, laboratory, and medication data are obtained from each site’s electronic health record (EHR) and other institutional data sources. CNICS study procedures have been approved by Institutional Review Boards at each participating site.

### Study Participants

We examined PWH in care at UNC and UW who had HIV VL and CD4 cell count measures between January 2015 - May 2023. We restricted our analysis to active participants, which we defined as having had a clinical visit in the 18 months prior to May 2023. We also excluded PWH who did not provide an address or whose formal address was either a PO box or general delivery address, as these types of addresses do not correspond to a physical location and therefore cannot be mapped to specific geographic coordinates. Participants experiencing homelessness or unstable housing who provided a valid address, such as an identifiable location or a shelter, were included in our study population.

### Geocoding Process

We collected the most recent street address for CNICS participants recorded in site registry systems linked to EHR databases. The routine updating of addresses assumes that the most recent address is also the most current address, because study participants had the opportunity to update their address at their clinical visit. We reviewed and manually cleaned addresses prior to geocoding, which included standardization, ensuring completeness, and correcting misspellings in city or street names and missing data (e.g., adding missing ZIP codes). Geocoding was performed using ArcGIS Pro and the ArcGIS World Geocoding Service. If automatic ArcGIS settings were unsuccessful in geocoding, interactive geocoding was performed, which involved manual processing, verifying, and correcting addresses. We excluded addresses with an ArcGIS geocoding match address score of < 80%. We accessed 2020 census tract cartographic boundary shapefiles from the US Census Bureau [33] and spatially joined them to the geocoded patient level address point file.

### Community Resilience Index

The Community Resilience Estimates (CRE) index is generated by the U.S. Census Bureau, Department of Commerce, based on 10 demographic, social, and housing characteristics that represent specific resilience-related social vulnerability components: 1) Income-to-Poverty Ratio is < 130%; 2) Single or zero caregiver household; 3) Unit level crowding with ≥ 0.75 persons per room; 4) Communication barrier, defined as either Limited English Speaking households OR no one in the household over the age of 16 with a high school diploma; 5) No one in the household is employed full-time, year-round; 6) Disability (difficulty with hearing, vision, cognitive, ambulatory, self-care, or independent living) posing constraint to significant life activity; 7) No health insurance coverage; 8) Being aged 65 years or older; 9) No vehicle access; 10) Households without broadband internet access [25]. 

The components utilized by the CRE, such as income-to-poverty ratio and health insurance coverage, impact the community’s ability to respond and recover from adversity. The CRE provides a metric to identify socio-economic vulnerabilities and measure how socially vulnerable communities within the US are to the impacts of disasters, including public health emergencies [25]. While the CRE does not measure community resiliency directly, it compiles components that may affect a community’s resiliency and thus identifies populations that are likely to be more impacted by and have greater difficulties overcoming disasters [25]. 

The CRE index is generated using microdata information on individuals and households from the 2022 American Community Survey (ACS) [34], the Census Bureau’s Population Estimates Program (PEP) [35], and the 2020 Decennial Census [36]. Each vulnerability is estimated using small area modeling techniques [37]. Individuals in the sample (ACS, PEP, and the 2020 Decennial Census) are assigned a binary value (0 for absent,1 for present) for each of the 10 components based on their individual and household attributes; for household-level variables, each individual in the household is assigned a risk factor if the household meets the criteria for that risk factor. The vulnerabilities are then summed to create the CRE index. The US Census Bureau categorizes the CRE index into three groups: 0 social vulnerability components, 1–2 social vulnerability components, and ≥ 3 social vulnerability components. Subsequently, each census tract is assigned to the percent of people living in that census tract that have 0 social vulnerabilities, 1–2 social vulnerabilities, and ≥ 3 social vulnerabilities. We then assigned each census tract in our study to the predominant CRE category that made up each census tract.

### HIV-related Clinical Outcomes

Our outcomes of interest were unsuppressed HIV VL defined as ≥ 50 copies/mL and low CD4 cell count defined as < 350 cells/mm^3^ using the most recent measure available for each participant between January 2015 - May 2023.

### Statistical Analysis

We conducted individual-level analyses of PWH in our study. We first calculated descriptive statistics to summarize the demographic and clinical characteristics of PWH by census tract CRE index category. We then estimated prevalence ratios (PR) and 95% confidence intervals (CI) for the relationship between CRE (considered as 0 [reference], 1–2, or ≥ 3 vulnerabilities) and our two outcomes: unsuppressed VL and low CD4 cell count, using modified Poisson [38] generalized estimating equations to account for clustering by census tract. Our primary analysis adjusted for individual-level age, sex, race/ethnicity, site, and year of outcome measurement. Additionally, we conducted two sensitivity analyses: (1) unadjusted and (2) site-stratified, utilizing the main multivariable model. Analyses were conducted using Stata version 17.0 (StataCorp, College Station, TX).

## Results

Among the 3,191 PWH in our geocoded study cohort, 2,575 (81%) were male and median age was 53 (interquartile range [IQR]: 41–61) (Table 1). Participants were racially and ethnically diverse, with 1,398 (44%) non-Hispanic White, 1,212 (38%) non-Hispanic Black, 377 (12%) Latinx/Hispanic, and 204 (6%) another or unknown race/ethnicity. Overall, 342 (11%) were virally unsuppressed and 528 (17%) had low CD4 cell count. The median year of laboratory measurements for VL and CD4 count was 2022 (interquartile range [IQR] = 2022–2023 and 2021–2023, respectively). A majority of participants (69%) lived in a census tract that predominantly had 1–2 social vulnerabilities, followed by 0 social vulnerabilities (27%), and ≥ 3 social vulnerabilities (5%); among participants living in census tracts categorized as having ≥ 3 social vulnerabilities, there were a higher proportion of participants from UNC compared to UW (54% vs. 46%, respectively).

**Table 1:** Demographic and clinical characteristics of people with HIV by census tract community resilience estimates (CRE) category, 2015–2023

	Total	Zero Social Vulnerabilities^1^	1–2 Social Vulnerabilities^1^	3 or More Social Vulnerabilities^1^
	*N* (%)	*N* (%)	*N* (%)	*N* (%)
	3191	852 (27) ^**2**^	2192 (69) ^**2**^	147 (5) ^**2**^
Age (Median, IQR)	53 (41–61)	53 (41–60)	53 (42–61)	53 (41–62)
Male	2575 (81)	721 (85)	1742 (80)	112 (76)
Race/Ethnicity				
Non-Hispanic White	1398 (44)	429 (50)	922 (42)	47 (32)
Non-Hispanic Black	1212 (38)	286 (34)	848 (39)	78 (53)
Latinx, Hispanic	377 (12)	90 (11)	270 (12)	17 (12)
Other, Unknown	204 (6)	47 (6)	152 (7)	5 (3)
**Site**				
University of North Carolina, Chapel Hill	1354 (42)	370 (43)	905 (41)	79 (54)
University of Washington, Seattle	1837 (58)	482 (57)	1287 (59)	68 (46)
Unsuppressed HIV viral load (≥ 50 copies/mL)	342 (11)	73 (9)	244 (11)	25 (17)
Low CD4 count (< 350 cells/mm ^3^ )	528 (17)	129 (15)	363 (17)	36 (24)

Abbreviations:* IQR* Interquartile Range,* CRE* Community Resilience Estimates

1 There are 10 resilience-related social vulnerabilities included in the CRE index: [1] Income-to-Poverty Ratio is < 130%; [2] Single or zero caregiver household; [3] Unit level crowding with ≥ 0.75 persons per room; [4] Communication barrier defined as either Limited English Speaking households OR no one in the household over the age of 16 with a high school diploma; [5] no one in the household is employed full-time, year-round; [6] Disability (difficulty with hearing, vision, cognitive, ambulatory, self-care, or independent living) posing constraint to significant life activity; [7] no health insurance coverage [8], being aged 65 years or older [9], no vehicle access [10], households without broadband internet access

2 Shown as row percentages

There were differences in participant characteristics between census tracts by predominant social vulnerability category. There was a lower proportion of males who lived in census tracts with predominantly ≥ 3 social vulnerabilities, compared to 0 social vulnerabilities, 76% vs. 85%, respectively (Table 1). Additionally, more non-Hispanic White (50%) and less non-Hispanic Black (34%) participants lived in 0 social vulnerability census tracts, compared to census tracts with predominantly ≥ 3 social vulnerabilities (32% non-Hispanic White, 53% non-Hispanic Black).

In our primary adjusted analysis, we found that PWH living in census tracts with predominantly 1–2 social vulnerabilities were 1.28 times more likely (95% CI: 1.00–1.63.00.63) to be virally unsuppressed compared to participants living in census tracts with predominantly 0 social vulnerabilities, while PWH living in census tracts with predominantly ≥ 3 social vulnerabilities were 1.95 times more likely (95% CI: 1.22–3.10) to be virally unsuppressed (Table 2). Additionally, compared to PWH living in census tracts with predominantly 0 social vulnerabilities, PWH living in a census tract with predominantly ≥ 3 social vulnerabilities were 1.51 times more likely (95% CI: 1.12–2.04) to have a CD4 count < 350 cells/mm^3^. We did not observe an association between PWH living in census tracts with predominantly 1–2 social vulnerabilities and low CD4 cell count. Results were similar in unadjusted analyses (Supplemental Table 1).

**Table 2:** Associations between census tract community resilience estimates (CRE) category and HIV health-related outcomes among people with HIV. (*N* = 3191)

	VL ≥ 50 copies/mL		CD4 < 350 cells/mm^3^	
CRE Category	PR (95% CI)	*p*-value	PR (95% CI)	*p*-value
0 social vulnerabilities (ref)	1.0	-	1.0	-
1–2 social vulnerabilities	1.28 (1.00–1.63.00.63)	0.05	1.04 (0.87–1.24)	0.6
≥ 3 social vulnerabilities	1.95 (1.22–3.10)	0.005	1.51 (1.12–2.04)	0.007

Abbreviations:* CRE* Community Resilience Estimates,* VL* Viral load,* PR* Prevalence ratio,* CI* Confidence interval

Estimates from generalized estimated equations clustered by census tract and adjusted for age, sex, race/ethnicity, site, and year of outcome measurement

Results from our site-stratified analysis were similar to the main analysis, however there was greater variability present in both the VL and CD4 estimates at higher levels of social vulnerabilities where sample sizes were small (Supplemental Table 2).

## Discussion

Our results demonstrate the relationship between community-level social vulnerabilities and unsuppressed VL and low CD4 counts among PWH. Our findings suggest that PWH living in census tracts with ≥ 3 social vulnerabilities are nearly two times more likely to have unsuppressed VL, and about 50% more likely to have low CD4 counts. Overall, we found that community-level factors, specifically social vulnerabilities, are associated with individual-level HIV-related health outcomes. These findings emphasize the need to focus on mitigating communities’ social vulnerabilities to improve PWH’s overall health.

Among PWH, VL is the core biomarker for monitoring HIV infection and progression [7]. Viral suppression is important for maintaining long-term health and limiting HIV transmission within the community [7]. In addition, CD4 count is commonly used to measure the progression of an individual’s HIV disease [39]. Low CD4 count is associated with higher likelihood of hospitalization [40, 41], greater vulnerability to opportunistic infections [42, 43], and increased risk of cancer [44] and mortality [45]. Our findings suggest that PWH in socially vulnerable areas are substantially more likely to have unsuppressed VL and low CD4, which translates to them being more at risk for systemic inflammation [46, 47] and immune activation [46], as well as worsening HIV-associated conditions (e.g., kidney disease [48] and tuberculosis [49]).

These findings are consistent with the growing body of literature demonstrating that PWH residing in areas characterized by higher social vulnerability experience elevated risks of poor HIV-related outcomes [24, 50, 51]. For example, past research found that the proportion of PWH who achieved viral suppression within 12 months of diagnosis increased as neighborhood poverty decreased [24]; conversely, PWH living in neighborhoods with higher poverty rates were more likely to have lower CD4 cell counts [52]. Another study found that high poverty, high alcohol outlet density, low car ownership, high number of bus stops, lower educational attainment, and greater percentage of vacant housing were associated with low levels of community viral suppression [53]. An increase in economic instability, lack of education, and being uninsured was also found to be associated with an increase in the number of PWH with unsuppressed VL [51]. Our findings further highlight that community-level social vulnerability is associated with the ability of PWH to achieve optimal HIV treatment outcomes.

Related research shows that social vulnerability may also affect incidence of HIV as well as diagnosis [1, 2] and/or initiation of care [2]. Studies have shown that people who live in communities with the highest social vulnerability are at increased risk for HIV infection [54]. Individuals who live in communities characterized by high levels of social vulnerability often face barriers to receiving HIV diagnosis and initiating treatment for HIV [2], including systemic inequalities and reduced access to healthcare [1, 2]. The risk of delayed diagnosis and treatment leading to longer period of active viremia and lower CD4 counts [55].

At the community level, resource accessibility [56] and social support [10, 57] impact a community member’s health-related options and decisions. Communities with greater resilience or low social vulnerabilities, characterized by strong social networks and robust infrastructures, may be better equipped to provide the support and resources necessary for PWH to manage their health effectively, promoting better health outcomes and overall well-being. Conversely, PWH living in communities with high social vulnerability may be exposed to numerous impediments to obtaining HIV care, including housing insecurity, HIV stigma, limited access to transportation, and poverty [58]. These structural barriers are compounded by factors such as discrimination and language or cultural barriers, significantly affecting a person’s quality of and access to care [1].

The geographic distribution of social vulnerability is likely not random and, in some areas, reflects a history of disinvestment [59]. Factors such as exclusionary housing practices and the exodus of white populations from cities to suburbs have resulted in the disproportionate concentration of minoritized groups in areas that continue to be characterized by adverse conditions, limited resources, and greater health risks [59, 60]. It is important to emphasize that PWH may live in census tracts with high social vulnerability because of these structural drivers of community level disparities [50, 61] that are associated with a higher risk of HIV incidence and poor health outcomes [62]. Additionally, some PWH may choose to live closer to their care provider [63], which in some cases may be located in areas with higher social vulnerability. This suggests a need for interventions that address structural barriers and improve healthcare access, economic stability, and social support for highly vulnerable communities [50, 64].

Policy and system level interventions can be effective solutions to address these community-level disparities. Evidence-based models such as Housing First and the Housing Opportunities for Persons With AIDS (HOPWA) have been shown to improve ART adherence [65, 66] and viral suppression rates [67]. Similarly, Medicaid expansion has been associated with increased access to HIV testing, diagnosis, and ART initiation [68]. These interventions demonstrate that shifting the focus from individual-level risk to community level conditions can yield equitable and cost-effective outcomes in HIV care [69].

Geocoded data allow for examination of the role that community-level social vulnerability may have on HIV-related health outcomes and suggest the potential for targeting resources and interventions based on community indicators. The CRE index is a social vulnerability measure that was created to identify communities that may be more impacted and have greater difficulty overcoming disaster-related events (e.g., natural disasters, economic shocks, public health pandemics, etc.) [37]. Importantly, the CRE highlights the inextricable relationship between resilience and vulnerability, whereby high levels of vulnerability can significantly erode resilience, leading to greater susceptibility to adverse health outcomes [70]. For example, in communities where a high proportion of members are uninsured, which is one of the 10 components of the CRE index, even those with insurance are significantly affected by other community members’ lack of insurance [71, 72]. These communities often lack robust healthcare infrastructure due to insufficient funding resources [73], making it difficult to provide adequate health care services to the community members [74, 75], especially in times of crises. This example underscores the impact of social vulnerabilities on a community by affecting its ability to provide robust care to all community members, including PWH, both during times of disasters and not.

In addition to the CRE, researchers have developed other area-level measurements to capture neighborhood socioeconomic conditions and disparities. These include various types of livability and opportunity indices, each aiming to assess specific community characteristics that support well-being and access to resources, and other spatial measures of social and economic stratification such as the Index of Concentration at the Extremes (ICE) [76]. While several area-level measures may be relevant to the health and well-being of PWH, the CRE offers distinct advantages for this study, due to its specific focus on social vulnerability factors relevant to public health emergencies and resilience to health shocks [77]. The CRE compiles 10 public health-related indicators (e.g. lack of health insurance or transportation) that could conceivably be directly intervened on to improve health. In contrast to related measures that use aggregate community data, such as the U.S. Centers for Disease Control and Prevention’s Social Vulnerability Index [3], the CRE’s use of Census microdata uniquely quantifies the burden of intersectional social vulnerabilities at the individual level [77, 78]. However, the CRE does not disaggregate which of the 10 social vulnerabilities are predominant in each community, limiting its utility for targeting specific factors. Overall, the CRE provides a novel, public health-oriented approach to quantifying social vulnerability, which makes it particularly effective in examining the relationship between place-based disparities and HIV-related outcomes.

This study was meant to be hypothesis generating; therefore, it highlights the need for future research focused on social vulnerability components to estimate the relationship between individual social vulnerabilities and HIV-related health outcomes. In addition, future research could study how social vulnerability affects HIV incidence and HIV outcomes across temporal and clinical spectra. To effectively address community-level social vulnerabilities, a multi-pronged approach between clinical and public health practice is required. Social vulnerability screenings may be incorporated as a part of routine clinical care to identify PWH at risk for poor health outcomes due to environmental or socioeconomic barriers, enabling targeted interventions. The examination of longitudinal trends will be critical to assessing how changes in social vulnerability over time affect HIV health outcomes, treatment adherence, and disparities in care. Ultimately, this research would aid in the development of targeted interventions aimed at reducing health disparities among PWH through modifiable social vulnerabilities at the community level. Implementing HIV interventions that place an emphasis on reducing disparities has the potential to improve public health for not only PWH, but also the community [1, 7, 79].

### Limitations & Strengths

This study should be interpreted in the context of several limitations. The cross-sectional nature of this study does not allow for causal inference. In addition, there are potential temporal limitations since we could not capture long-term trends between community resilience and HIV-related health outcomes. The process of geocoding requires a valid address, which may exclude specific demographic groups, such as people experiencing homelessness. There is also potential limited generalizability of our study since the study population was PWH receiving at least some amount of HIV clinical care, which limits the sample to PWH with a higher healthcare utilization rate. We expect that these limitations exclude more vulnerable individuals who would have even stronger associations between community-level social vulnerabilities and HIV-related outcomes (i.e., individuals in areas with high social vulnerability could have more difficulty accessing care and achieving optimal HIV-related outcomes). In addition, this study only included two geographic areas; associations with community resilience may differ in other areas. There are also limitations associated with the CRE, including that the CRE was developed to measure vulnerability to disasters and public health emergencies, rather than a direct proxy for social determinants of health. The CRE also does not account for community resilience factors that could mitigate the impact of the social vulnerabilities experienced by the community. Additionally, the CRE could lead to misinterpretation by placing the responsibility for community-level social vulnerability on the individual, rather than on the structural factors that created these conditions. Finally, in our analysis we assigned a single predominant category for each census tract, which may not fully reflect the heterogeneity of vulnerabilities within neighborhoods.

Despite these limitations, our study exhibits several notable strengths. We utilize the CRE to assess social vulnerabilities at the community level, which offers a novel framework for understanding HIV-related health outcomes. Our study also employed census tract level geocoded data, which is a more granular geographic area than research conducted on community characteristics and HIV-related health outcomes at the ZIP Code or county level. Additionally, this study utilizes geocoded individual-level clinical data from a large, diverse cohort of PWH across heterogenous geographic areas. This data enabled us to demonstrate that the social vulnerabilities of communities remain an important factor in HIV-related health outcomes even after accounting for individual-level characteristics that may differ between places. Data from multiple sites also enabled us to show that community social vulnerability plays an important role in different places, while the strength and magnitude of these relationships likely differs by community. Future analyses with larger sample sizes should conduct heterogeneity analyses by site to better understand the nuanced relationship between community social vulnerability and site.

## Conclusion

Overall, these findings underscore the intricate relationship between community-level social vulnerability and HIV-related health outcomes among PWH in two geographic areas. Our study used individual-level clinical data and identified an association between social vulnerability and unsuppressed VL and low CD4 cell counts in PWH. Addressing social vulnerabilities within a community could potentially improve the treatment adherence and clinical outcomes of PWH, which has implications for preventing hospitalizations, opportunistic infections and other diseases or death. These results emphasize the importance of acknowledging community health to improve individual health, especially in relation to HIV well-being. In the context of PWH, our findings suggest that the CRE are a tool that could be used to highlight areas for targeted interventions based on community-level influences to improve HIV-related outcomes. Our study also contributes to the growing body of research that supports the need for HIV interventions focused on reducing health disparities.

## Electronic Supplementary Material

Below is the link to the electronic supplementary material.

Supplementary Tables 1 and 2
