# Supplementary material for: Community Resilience Estimates and HIV Health Outcomes: Insights from the CNICS Cohort
Source: AIDS Behav. 2025 Dec 16;30(5):1516–25. doi: 10.1007/s10461-025-04984-5 (PMC13167882; doi:10.1007/s10461-025-04984-5)
Supplement: Supplementary file 1 — Supplementary Tables 1 and 2 [file 10461_2025_4984_MOESM1_ESM.docx]

**Supplemental Table 1.** Unadjusted associations between census tract community resilience estimates (CRE) category and HIV health-related outcomes among people with HIV, clustering by census tract.

|  | **VL ≥50 copies/mL** | | **CD4 <350 cells/mm^3^** | |
| --- | --- | --- | --- | --- |
| **CRE Category** | **PR (95% CI)** | **p-value** | **PR (95% CI)** | **p-value** |
| 0 social vulnerabilities (ref) | 1.0 |  | 1.0 |  |
| 1-2 social vulnerabilities | 1.29 (1.00-1.68) | 0.04 | 1.09 (0.89-1.34) | 0.4 |
| ≥3 social vulnerabilities | 1.98 (1.14-3.47) | 0.02 | 1.62 (1.11-2.35) | 0.01 |

Abbreviations: CRE, Community Resilience Estimates; VL, viral load; PR, prevalence ratio; CI, confidence interval.

**Supplemental Table 2.** Associations between census tract community resilience estimates (CRE) category and HIV health-related outcomes among people with HIV stratified by site.

|  | **VL ≥50 copies/mL** | | **CD4 <350 cells/mm^3^** | |
| --- | --- | --- | --- | --- |
|  | **PR (95% CI)** | **p-value** | **PR (95% CI)** | **p-value** |
| **University of Washington, Seattle** | | | | |
| **CRE Category** |  |  |  |  |
| 0 social vulnerabilities (ref) | 1.0 |  | 1.0 |  |
| 1-2 social vulnerabilities | 1.24 (0.90-1.71) | 0.18 | 0.96 (0.78-1.18) | 0.7 |
| ≥3 social vulnerabilities | 2.51 (1.56-4.02) | <0.001 | 1.65 (1.21-2.26) | 0.001 |
| **University of North Carolina, Chapel Hill** | | | | |
| **CRE Category** |  |  |  |  |
| 0 social vulnerabilities (ref) | 1.0 |  | 1.0 |  |
| 1-2 social vulnerabilities | 1.34 (0.90-1.98) | 0.14 | 1.21 (0.87-1.70) | 0.27 |
| ≥3 social vulnerabilities | 1.36 (0.66-2.79) | 0.41 | 1.33 (0.77-2.29) | 0.31 |

Abbreviations: CRE, Community Resilience Estimates; VL, viral load; PR, prevalence ratio; CI, confidence interval.

Estimates from generalized estimated equations clustered by census tract and adjusted for age, sex, race/ethnicity, site, and year of outcome measurement.
